# EVITA: a tool for the early EValuation of pharmaceutical Innovations with regard to Therapeutic Advantage

**DOI:** 10.1186/1472-6904-10-5

**Published:** 2010-03-16

**Authors:** Isabel Püntmann, Norbert Schmacke, Arne Melander, Gunnar Lindberg, Bernd Mühlbauer

**Affiliations:** 1 Department of Pharmacology, Klinikum Bremen-Mitte gGmbH, Bremen, Germany; 2 Working Group Health Services Research, University of Bremen, Bremen, Germany; 3 The Nepi Foundation and Department of Clinical Sciences, Lund University, Clinical Research Centre, Malmö University Hospital, Malmö, Sweden

## Abstract

**Background:**

New drugs are generally claimed to represent a therapeutic innovation. However, scientific evidence of a substantial clinical advantage is often lacking. This may be the result of using inadequate control groups or surrogate outcomes only in the clinical trials. In view of this, EVITA was developed as a user-friendly transparent tool for the early evaluation of the additional therapeutic value of a new drug.

**Methods:**

EVITA does not evaluate a new compound *per se *but in an approved indication in comparison with existing therapeutic strategies. Placebo as a comparator is accepted only in the absence of an established therapy or if employed in an *add-on *strategy on top. The evaluation attributes rating points to the drug in question, taking into consideration both therapeutic benefit and risk profile. The compound scores positive points for superiority in efficiency and/or adverse effects as demonstrated in randomized controlled trials (RCTs), whilst negative points are awarded for inferiority and/or an unfavorable risk profile. The evaluation follows an algorithm considering the clinical relevance of the outcomes, the strength of the therapeutic effect and the number of RCTs performed. Categories for therapeutic aim and disease severity, although essential parts of the EVITA assessment, are attributed but do not influence the EVITA score which is presented as a color-coded bar graph. In case the available data were unsuitable for an EVITA calculation, a traffic-type yield sign is assigned instead to criticize such practice. The results are presented online http://www.evita-report.de together with all RCTs considered as well as the reasons for excluding a given RCT from the evaluation. This allows for immediate revision in response to justified criticism and simplifies the inclusion of new data.

**Results:**

As examples, four compounds which received approval within the last years were evaluated for one of their clinical indications: lenalidomide, pioglitazone, bupropion and zoledronic acid. Only the first achieved an EVITA score above zero indicating therapeutic advantage.

**Conclusions:**

The strength of EVITA appears to lie in its speedy assessment of the potential therapeutic advantage of a new drug for a given indication. At the same time, this approach draws attention to the typical deficits of data used for drug approval. EVITA is not intended to replace classical health technology assessment reports but rather serves as a screening tool in the sense of horizon scanning.

## Background

Every year, numerous new drugs are introduced into the pharmaceutical markets worldwide. In general, the therapeutic efficiency of a drug is not satisfactorily defined at the time of its approval by the health authorities. For decades, a new pharmacological approach or a new molecular structure, i.e. new chemical entity (NCE), *per se *justified the claim that a compound represented a pharmaceutical innovation. Today, however, a new compound must exhibit a clinically relevant advantage over the existing established therapy in order to be considered an innovation. Comprehensive health technology assessments such as reports by the British National Institute for Clinical Excellence (NICE) or the German Institut für Qualität und Wirtschaftlichkeit im Gesundheitswesen (IQWiG) are, without doubt, the benchmark for such evaluations but they are time-consuming. Common evaluation strategies, such as the "A through D" classification used in Germany established by Fricke and Klaus [1] are still based on the NCE principle and thus simplify the result. More sophisticated evaluation strategies like the one described recently by Caprino and Russo [2] are well designed but appear to be too complicated to be carried out by persons not specialized in drug efficiency evaluation.

However, there is a clear lack of a transparent and user friendly tool to answer the simple question of whether a new compound offers an additional therapeutic benefit over existing therapeutic strategies for a given medical situation. This project has developed an algorithm for the early evaluation of the risk-benefit ratio of new drugs, EVITA (**EV**aluation of pharmaceutical **I**nnovations with regard to **T**herapeutic **A**dvantage). The additional therapeutic benefit of a drug is assessed by taking into account four considerations, a) therapeutic aim, b) disease category, c) trial setting and, most important, d) a score calculated by a benefit/risk consideration. EVITA is intended to be used by physicians and other professionals in the health sector, who are concerned with innovations in pharmacotherapeutics. EVITA is easy to use, however, basic knowledge in critical appraisal is a precondition.

## Methods

### Principle

Since therapeutic efficiency cannot be defined in general terms but only in respect of a specific medical situation, the EVITA algorithm can only be used to evaluate a new compound for a given indication. EVITA attributes positive or negative rating points to the drug in question and its comparator in clinical trials by assessing both therapeutic benefit and risk profile, i.e. rate and strength of adverse effects attributed to the drug.

EVITA is not intended to generate an absolute score value solely but a benefit/risk score annotated with specific information on therapeutic aim, disease category and trial setting. The therapeutic aim, distinguishing treatment from prevention, has an impact on the EVITA score (see below) (Table 1 + 2). Disease severity, however, graduated in four categories from reversible and moderate discomfort to life-threatening symptoms, has no effect on the EVITA score even though it is essential when considering the benefit of a drug as opposed to its risk profile. However, such appraisal appears to be rather subjective, if not arbitrary. Thus, the severity of the disease is categorized and documented but is not a variable in the calculation (Table 1).

**Table 1:** Therapeutic Aim and Disease Category

prevention	to reduce risk of disabling or imparing events
**treatment**	to cure diseases, to substitute missing substances
	indispensable to life, to modify or relieve symptoms
	**severity grading of the diseases:**
	I. acute life-threatening or severe chronic disease
	II. rehabilitation
	III. less severe acute or chronic disease
	IV. application outside a treatment context

Therapeutic Aim and Disease Category according to disease severity

**Table 2:** Modifier

prevention		
**NNT**	**ARR**	**Modifier**

<20	5-100%	2.0
20 - <50	<5%	1.75
50 - <100	<2%	1.5
100 - <175	<1%	1.25
175 - <300	<0.57%	1.0
300 - <500	<0.33%	0.75
500 - <1000	<0.2%	0.5
≥1000	<0.1%	0.25

**treatment**		

**NNT**	**ARR**	**Modifier**

<3	>30%	2.0
3 - <10	10-30%	1.5
≥10	<10%	1.0

Assessment of the modifier for the efficiency score according to strength of effect given in "number needed to treat" (NNT) or "absolute risk reduction" (ARR).

### Trial settings

**Figure 1:**
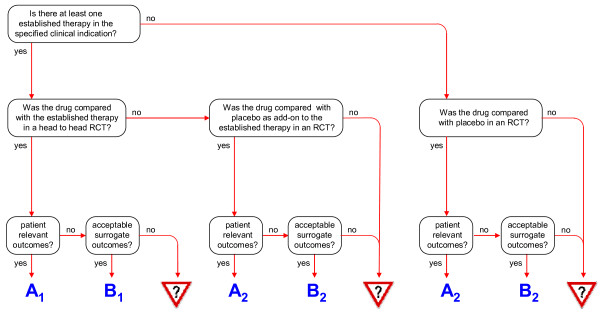
**Flowchart showing the decision tree leading to the EVITA trial settings**.

EVITA assessment initially defines the conditions which must be met for the randomized controlled trials (RCTs) to be included in the evaluation process. The decision to include or exclude an RCT depends on the one hand on the presence or absence of an established therapy and on the other hand on the type of outcome variables employed in these studies. As a minimum requirement for an RCT to be taken into consideration for EVITA evaluation, a Jadad Score of at least 3 has to be met [3]. The respective decision pathway is outlined in the form of a flowchart in Figure 1. As a result of the decision-making tree, different "trial settings" of EVITA can be defined.

Trial setting A represents clinical research which addresses patient relevant outcomes including mortality, reduced progression rate, event reduction, restoration or preservation of functionality, control of symptoms and quality of life. Within this A level, trial setting A_1 _applies to testing the potential superiority of a new drug over established therapy by one or more *head-to-head *RCTs. The clinically relevant therapeutic benefit can also be tested by comparison with placebo either directly, if there is no established therapy, or administered on top of a given established therapy, i.e. the *add-on *situation (trial setting A_2_). If new treatment strategies are investigated with respect to acceptable surrogate outcomes only, trial setting B is attributed, with B_1 _for the *head-to-head *situation and B_2 _for the placebo comparison (including *add-on *situations), as described for the A trial settings. In situations with insufficient trials for a justified EVITA calculation the trial setting is defined as N/A (EVITA calculation **n**ot **a**vailable).

### Point scoring for efficiency, risk profile, and the EVITA score

The compound of interest gains positive score points if it is shown to be superior to the current established therapy. In the absence of such established therapy, superiority over placebo can be interpreted as therapeutic benefit. Furthermore, the compound of interest receives a positive rating if the investigational drug demonstrates a more favorable risk profile than its comparator.

Conversely, negative rating points are attributed to the drug if the clinical trials result in inconclusive data, lack of superiority or even proof of inferiority and/or a less favorable risk profile than the comparator. Both the benefit and the risk profile of the drug are evaluated on the basis of RCTs.

The point scores for efficiency are modified by the number of clinical trials, the trial setting encountered and the therapeutic aim the agent is used for (Table 2 and 3). Superiority or inferiority proven by two or more studies result in a higher positive or negative score than proof from only one study (Table 3). Studies demonstrating non-inferiority or equivalence of a new drug compared with the given established therapy have to be considered in a more differentiated way. In the case of trials designed to demonstrate the superiority of an investigational agent, a resultant positive score will be reduced by tests showing non-inferiority or equivalence. Conversely, in the case of inferiority trials, a resultant negative score will be attenuated in favor of the investigational agent by further studies showing non-inferiority or equivalence. In respect of the superiority or inferiority, two or more non-inferiority/equivalence trials will have a higher numeric impact than only one (Table 3).

**Table 3:** **Efficiency Profile**

RCTs showing evidence of	number of RCT	patient relevant outcome	surrogate outcome
	**0**	**0**	**0**
**superiority**	**1**	**+5.0**	**+2.5**
	**≥2**	**+7.5**	**+3.75**

	**0**	**0**	**0**
**non-inferiority/equivalence ** (in the presence of other RCT showing superiority)	**1**	**-1.67**	**-0.83**
	**≥2**	**-2.5**	**-1.25**

**non-inferiority/equivalence ** (in the absence of other RCT)	**any**	**0**	**0**

	**0**	**0**	**0**
**non-inferiority/equivalence ** (in the presence of other RCT showing inferiority)	**1**	**+1.67**	**+0.83**
	**≥2**	**+2.5**	**+1.25**

	**0**	**0**	**0**
**inferiority**	**1**	**-5.0**	**-2.5**
	**≥2**	**-7.5**	**-3.75**

**sum**		**...**	

**modifier**		**...**	

**efficiency score**		**...**	

Efficiency score according to number of RCTs and outcome variables investigated as well as modification by strength of effect as extracted by the modifier assessment (Table 2).

Point scores are multiplicated by two when evidence is drawn from trials investigating patient-relevant outcomes (trial setting A) than from trials investigating surrogate outcomes only (trial setting B) (Table 3).

The efficiency score also takes into account the strength of the therapeutic effect, the measure of which is the number needed to treat (NNT). As can be seen from Table 2, the modification of the efficiency score due to the therapeutic strength is differentiated according to the treatment goals. For preventive situations, the NNTs may increase the benefit score, leave it unchanged or even reduce it, which is reflected in the requirement of prudently low NNTs to outweigh the potentially negative consequences of long-term drug therapy. In contrast, drugs for the therapy of acute disorders with treatment aims such as healing and symptom relief would, in general, have low NNTs. In this therapeutic situation it seems sufficient to have a positive correction factor only for agents with a particularly low NNT (Table 2).

The risk score of an investigational drug is based on information about adverse effects and interactions with other interventions. To ensure a balanced evaluation and a justified comparison with the established therapy or placebo, only data from controlled trials is included. The adverse effects are scored on the basis of severity and frequency (Table 4). EVITA considers the highest occurrence of adverse effects (AE) from each of the three severity groups Grades 4 and 5 (disabling AE or life-threatening AE or death related to AE), Grade 3 (severe and undesirable AE) and Grades 1 and 2 (mild AE or moderate AE) according to the Common Terminology Criteria for Adverse Events (CTCAE Version 3.0, 2006) [4]. CTCAE grades are accepted criteria for the evaluation of adverse effects in clinical studies, including oncology trials. Frequency is evaluated according to the guideline on Summary of Product Characteristics (SPC) [5] in the following classes: rare/very rare (below 0.1%), less common (0.1 through below 1.0%), common (1.0 through below 10.0%), and very common (10.0% and above). It should be noted that very rare adverse effects (< 0.01%) cannot usually be detected in clinical trials because of limited subject numbers. Therefore, this frequency is taken together with the frequency class rare.

**Table 4:** Risk-Profile

severity grading	frequency	therapy investigated	therapeutic standard
**adverse events (AE)**			

**grades 5 + 4**	≥10%	**-4.0**	**-4.0**
(death related to AE or life-threatening AE or disabling AE)	≥1%	**-3.0**	**-3.0**
	≥0.1%	**-2.0**	**-2.0**
	<0.1%	**-1.0**	**-1.0**
	0	**0**	**0**

**grade 3 **	≥10%	**-2.5**	**-2.5**
(severe and undesirable AE)	≥1%	**-2.0**	**-2.0**
	≥0.1%	**-1.0**	**-1.0**
	<0.1%	**0**	**0**
	0	**0**	**0**

**grades 2 + 1**	≥10%	**-1.5**	**-1.5**
(moderate AE or mild AE)	≥1%	**-1.0**	**-1.0**
	≥0.1%	**-0.5**	**-0.5**
	<0.1%	**0**	**0**
	0	**0**	**0**

**Interactions**			

frequent or serious clinical consequence		**-2.0**	**-2.0**
occasional or may have clinical consequence		**-1.5**	**-1.5**
dose change		**-1.0**	**-1.0**
unlikely/probably or no clinical consequence		**0**	**0**
no information available		**-1.0**	**-1.0**

**sum**		**...**	**...**

**risk score**		**...**	

Risk Score Assessment. The severity grading is carried out according to the Common Terminology Criteria for Adverse Events version 3.0 (CTCAE v3.0) [4], the frequency according to the Guideline on Summary of Product Characteristics (SPC) [5]

The risk profile of an investigational drug cannot be evaluated in isolation. It rather has to be compared with that of the therapeutic alternative or the basal frequency of adverse effects of the underlying disease. Therefore, the score points for the risk profile are opposed to those observed in the control groups in the clinical trials, either the established therapy or placebo if applicable. Thus, the *eo ipso *negative value of the risk score of the investigational drug can be counterbalanced or even reversed to give a positive value if the adverse effects of the comparator dominate.

Interactions, mainly drug-drug-interactions, are a potential source of risk which can emanate from a drug. Since drug interactions are scarcely registered in clinical trials, this information is taken from the SPC.

The final stage of EVITA is adding up the efficiency and the risk score, both of which are evaluated in comparison to the established therapy, to obtain the EVITA score. Values considerably above zero indicate a likely potential that the new drug features a clinically relevant improvement in treatment; values around zero indicate an ambiguous state which might be clarified by further clinical trials.

It is unlikely that a drug will be introduced into the pharmaceutical market if its inferiority has been proven by two or more RCTs investigating patient relevant outcomes (-7.5) (Table 3) under established therapy or placebo in a preventive situation (modifying factor 2.0) (Table 2), and with an unfavorable risk and interaction profile in all severity groups (-8.0 and -2.0, respectively). Therefore, the lowest calculable EVITA score of -25.0, is not to be expected and should not arise. On the other hand, the maximum calculable EVITA score of +25.0 also appears unlikely since it would result from proof of superiority over the comparator in a treatment situation in at least two RCTs investigating patient relevant outcomes (+7.5) with low NNT (modifying factor 2.0) and a complete absence of adverse effects and interactions, while the comparator featured an unfavorable risk and interaction profile. Scores in the range of -5 to +10 are therefore to be expected as the typical EVITA result. It should be noted that EVITA may demonstrate an additional therapeutic benefit of a new compound despite a lack of superior efficiency because of a better risk and interaction profile.

**Figure 2:**
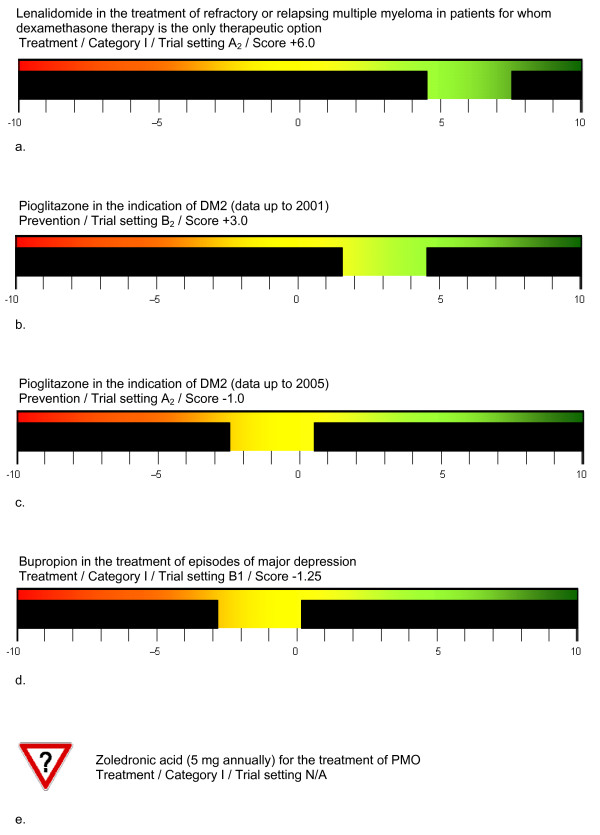
**Visualizations of the EVITA evaluations of the examples given in the present publication**. PMO, postmenopausal osteoporosis; DM2, diabetes mellitus type 2; see text for definition of the specific EVITA terms.

Since exact score values might suggest a more precise evaluation than EVITA can provide, the EVITA score is displayed as a color-coded bar graph, with green (positive values) indicating a compound that is likely to be innovative, yellow (values around zero) indicating an unclear result at the time and red (negative values) indicating an unlikely probability that the compound will offer an additional therapeutic benefit (Figures 2a-d). Further, a traffic-type yield sign incorporating a question mark is attributed for situations in which available data are unsuitable for an EVITA calculation indicating N/A trial settings (Figure 2e).

Transparency is a major concern of the EVITA project. To accomplish this, EVITA evaluation results are presented online http://www.evita-report.de together with the complete list of the source RCTs and the reasons for excluding any particular RCT from the evaluation. This also allows for rapid revision in response to justified criticism and simplifies the inclusion of new data e.g. from a new RCT publication.

## Results

### Lenalidomide for the treatment of refractory or relapsing multiple myeloma

Lenalidomide (Anatomic Therapeutic Chemical (ATC) Classification: L04AX04), a structural analogue to thalidomide, was introduced in 2007 (Germany), in combination with dexamethasone for the treatment of refractory or relapsing multiple myeloma (International Classification of Diseases Version 10 (ICD-10): C90) after at least one completed previous therapy [6,7]. Numerous therapeutic approaches are currently considered established treatment alternatives for this situation: high-dose dexamethasone, anthracycline ± vincristine-containing chemotherapeutic schemes, bendamustine, cyclophosphamide, bortezomib, or a second autologous stem cell transplantation can be taken as established therapy for this situation [8-11]. Without doubt, the therapeutic aim is "treatment" of a life-threatening disease indicating disease category I.

Two studies were found which met the EVITA requirements [12,13]. However, since both trials meticulously used the identical study protocol, were performed in the identical number and profile of patients and during the same time period, they have to be considered as one clinical trial. All patients received high dose dexamethasone and were randomly assigned to either additional lenalidomide or placebo.

Outcome was primarily measured using time to progression (TTP). Strictly speaking, this variable is a surrogate outcome. However, since, the overall survival (OS), measured as a secondary outcome, showed a clear tendency in favor of the study drug in this trial, TTP was accepted as a clinically relevant outcome. Thus, EVITA trial setting A_2 _applies, because lenalidomide was compared with placebo in an *add-on *situation with an established therapy using patient relevant outcomes (Figure 1). In the light of further established therapeutic alternatives the conclusion must be restricted to patients for whom dexamethasone appeared as the only therapeutic option.

The statistically significant outcome advantage demonstrated in one study attributes +5.0 points to lenalidomide (Table 3). As no NNT calculation is possible for the variable TTP, the higher proportion of patients, by over 38%, with an overall response was used instead, assuming a positive correlation between these variables. The NNT of 2.6 in 1.5 years (indicating 4 per year) results in a modifier factor of 1.5 (Table 2). The resulting efficiency score is +7.5.

The risk profile was calculated on the basis of observations of the adverse effect in the clinical trials used for the efficiency evaluation. There was a very similar frequency of adverse effects in the two treatment groups. Only grade 3 severity group adverse effects occurred slightly less frequently in the placebo group; in addition, there is a clinically relevant drug interaction risk, while for placebo interaction potential must be considered zero. The resultant risk score for lenalidomide is -1.5 (Table 4).

Taking efficiency and risk score together, gives an EVITA score of +6.0, indicating a rather likely chance that lenalidomide represents a patient relevant innovative drug for the specific subgroup of patients investigated in the trials cited (Figure 2a).

### Pioglitazone in the treatment of diabetes mellitus

Pioglitazone (ATC: A10BG03) is a thiazolidindione in the treatment of diabetes mellitus type 2 (DM2) (ICD-10: E10-E14) [14]. The evaluation was carried out twice, once based on the knowledge available in 2001, i.e. shortly after drug approval, and the other based on the knowledge of 2005, i.e. after publication of the *PROactive *study (*PROspective pioglitAzone Clinical Trial In macroVascular Events*) [15], a large outcome trial in diabetic patients.

Oral antidiabetics seek to protect the patient from macro- and microvascular sequelae, thus the therapeutic aim is "prevention". There are widely accepted established oral antidiabetic therapies, mainly using metformin and sulphonylurea compounds. In the pivotal studies [16,17], pioglitazone was tested in an *add-on *trial setting, metformin or sulphonylureas being employed as basal therapy. The outcome variable was glycosylated hemoglobin A1c (HbA1c), i.e. a surrogate variable. In view of the fact that the therapeutic efficiency of alternative oral antidiabetics, mainly metformin, was assured by clinical trials using patient relevant outcomes, a trial using surrogates only should no longer be accepted according to the EVITA evaluation strategy. However, since metformin was given as a basal drug in both groups, the therapeutic benefit of other antidiabetics on top of metformin is not satisfactorily ensured, and lowering blood glucose in diabetic patients is widely accepted as a necessary therapeutic goal, the trials using HbA1c as primary efficacy outcome were accepted. Hence, EVITA trial setting B_2 _applies (Figure 1).

For HbA1c, pioglitazone showed significant benefit over placebo in two trials, resulting in an efficiency score of +3.75 points, rounded to +4.0 (Table 3). The NNT for such a benefit cannot be calculated from this outcome. Therefore, since no other outcomes closely associated with the primary outcome were reported in this study the efficiency score was not modified further. The risk profile evaluation taken from the two trials showed an occurrence of less than 10% of both severe (-2.0 points) and mild to moderate (-1.0 points) adverse effects, but this applied to both the pioglitazone and the placebo treatment arm, so that the net risk for pioglitazone resulted in zero. According to the SPC, severe interactions with gemfibrozil and rifampicin may require dose changes of pioglitazone for which one negative score point is given, while for the comparator (placebo) a zero interaction potential is assumed (Table 4). The efficiency point score is +4.0 and the risk score -1.0, resulting in an EVITA score of +3.0 points which promises an additional therapeutic value of this new pharmacological candidate (Figure 2b).

Five years after approval, the results of the *PROactive *study were published [15]. In this trial, the effects of pioglitazone on the macrovascular outcome of over 5,000 DM2 patients treated with pioglitazone or placebo, both on top of established therapy, were investigated. The primary outcome - a composite of seven clinical events - is considered to be patient relevant so that EVITA trial setting A_2 _applies, i.e. a comparison of the patient relevant outcomes of an investigational drug with those of a placebo in an *add-on *situation (Figure 1). This trial failed to show the superiority of pioglitazone over placebo. According to the EVITA strategy, RCTs with patient relevant outcomes rule out trials with surrogate variables. Thus, unlike the earlier evaluation, the efficiency score decreases to zero (Table 3). Even if the individual risk profiles, as extracted from the Dormandy study, were changed from -7.5 points to -6.5 points, the reconciliation of both risk profiles would remain the same (-1.0 points) (Table 4) and the EVITA score would become negative (-1.0 points) (Figure 2c). According to the EVITA philosophy, point scores around zero are considered inconclusive for the interpretation of the innovative value of a new pharmacological candidate. Thus the aforementioned promise of an additional value is negated.

It should be noted that in the same publication [15] the authors communicated an additional outcome variable comprising only three of the seven original outcome components. This new outcome variable showed a statistically significant superiority of pioglitazone over placebo. However, since this variable was not mentioned in the original protocol [18], it cannot be considered predefined and thus cannot be included in the EVITA evaluation.

### Bupropion in the treatment of episodes of major depression

Bupropion (ATC: N06AX12, N07BA02) is a reuptake inhibitor of norepinephrine, dopamine, and, to a lesser degree, serotonine; it is also a nicotinic receptor antagonist. Originally marketed in the United States in 1984, bupropion was not introduced as an antidepressant in Germany until 2007 [19,20]. Major depression (ICD-10: F32-F39) is a severe if not life-threatening disease due to its elevated risk of suicide. Therefore category I applies. Although prevention is one aspect of antidepressive therapy, its main focus in the majority of patients is on the reduction of disease burden, thus defining the therapeutic aim as "treatment".

Bupropion has been on the pharmaceutical market for over two decades, so there is a plethora of clinical trial information. The majority of the older studies do not, however, meet the quality requirements of modern trial methodology such as conformity with Good Clinical Practice (GCP) or they focused on side effects such as disorders in sexual function as primary outcome. Therefore, they are unsuitable for EVITA evaluation of the therapeutic efficiency of bupropion. Some of the recently published studies [21-23] used placebo as the comparator. This cannot be accepted in indications where established therapies exist. Thus, only a minority of trials met the requirements for EVITA evaluation.

To fully assess all studies of antidepressant efficiency with an acceptable comparator, not only the trials published in peer-reviewed scientific journals were inspected, but also those published as abstracts on the company website, "GlaxoSmithKline (GSK) Clinical Trial Register". Finally, three trials were identified as suitable for EVITA evaluation. In a six-week study of 100 depressed patients [24], bupropion did not show superiority with respect to the Hamilton-scale (HAMD-17) over paroxetin, an antidepressant acceptable as established therapy. In two three-arm studies, only available online in the GSK Clinical Trial Register [25,26], bupropion was compared to placebo and venlafaxin using the "Montgomery-Asberg Depression Rating Scale"(MADRS) measuring changes after 8 or 10 weeks. The two studies showed divergent results. In one [25], while patients treated with bupropion showed a larger MADRS-score reduction than those under placebo, there was no difference in comparison to venlafaxine. In the other trial [26] bupropion induced similar MADRS-score reductions compared to placebo, albeit a smaller one when compared to venlafaxin.

Since each of the three trials represents a direct comparison with an established therapy and since scale-scores which quantify the severity of a disease generally have to be considered as surrogate, EVITA trial setting B_1 _applies (Figure 1). Therapeutic superiority over established therapy was not seen in any trial (0); however, inferiority in one study (-2.5) and equivalence in two studies (+1.25), resulted in a efficiency score of -1.25 which does not need to be adjusted for treatment strength (Table 3).

Due to the lack of complete publication of the three studies taken for the efficiency evaluation, there was insufficient information on the adverse effects profile of bupropion. Therefore, the respective SPC information was used to evaluate the risk profile. For comparison, the risk profile information in the SPCs of venlafaxin and paroxetin were considered. Since all compounds have been on the market for many years now, this procedure appeared to be justified. Although not all adverse effects (AE) were attributable to all the compounds, bupropion and its comparators paroxetin and venlafaxin, featured similar AE frequencies in all severity groups. While taking these antidepressants, patients experienced mild AE or moderate AE (Grades 1 and 2) very commonly, severe and undesirable AE (Grade 3) very commonly, and disabling AE or life-threatening AE or death related to AE (Grades 4 and 5) less commonly. Drug interactions occur occasionally and can also lead to clinical consequences during treatment with all of the three compounds. Thus, a point score of (-1.5) + (-2.5) + (-2.0) + (-1.5) = (-7.5) was calculated for the risk profiles of both bupropion and its comparators (venlafaxin and paroxetin), resulting in a risk score of zero for bupropion (Table 4).

The EVITA score for bupropion, obtained by adding up the -1.25 points for efficiency and the zero value for the relative risk profile was -1.25, indicating that an additional therapeutic benefit for bupropion in the treatment of major depression can hardly be expected (Figure 2d).

### Zoledronic acid (5 mg annually) for the treatment of postmenopausal osteoporosis (PMO)

Zoledronic acid (ATC: M05BA08) is a nitrogen-containing bisphosphonate which inhibits osteoclast-mediated bone resorption [27,28]. In Europe, intravenous zoledronic acid (4 mg administered every 3 to 4 weeks) was approved in 2001 for the prevention of skeletal complications in patients with bone metastases and for the treatment of tumor-induced hypercalcaemia [29]. It was introduced in 2005 as an annual intravenous formulation (5 mg/y) for the treatment of Morbus Paget [30]. In 2007, it was approved in the same dosage for the treatment of patients with PMO on high risk of fracture [31,32] (ICD-10: C80-C81), for which many other intravenous as well as oral bisphosphonates have been granted market authorization. PMO is a severe disease due to its elevated risk fractures, which if affecting the hip might be life-threatening. Therefore "treatment" and "category 1" apply for therapeutic aim and disease severity, respectively. From evidence-based perception, bisphosphonates, particularly alendronic and risedronic acid, raloxifene and estrogenes, are considered established therapies in the treatment of osteoporosis, as they have been proven to reduce the occurrence of clinically relevant bone fractures.

Two studies of zoledronic acid (5 mg once per year) treatment of PMO have been published so far. A dose-efficiency trial [33] investigated the influence on bone mineral density, a surrogate outcome. The pivotal trial *HORIZON *(*Health Outcomes and Reduced Incidence with Zoledronic Acid Once Yearly*) [34] investigated two primary efficiency outcomes, one of surrogate quality (risk reduction of morphometric vertebral fracture) and one of patient relevant quality (risk reduction of hip fracture). However, both trials were conducted in comparison with placebo. Against the background of existing established therapies, these studies did not fulfill the requirements for EVITA evaluation. As a consequence, zoledronic acid (5 mg annually) in the indication PMO had to be rated N/A (Figure 2e).

## Discussion

The main innovation of EVITA is to challenge the value of innovation *per se*. Previously, a new drug development was considered innovative when it featured a new chemical entity or had an effect on new pharmacological targets such as receptors, enzymes and signaling or transcriptional proteins. There is an abundance of drugs which after approval, in spite of positive initial expectations, have failed to demonstrate a clinically relevant benefit in controlled study situations or under everyday conditions. Well-known examples are flecainide [35], troglitazone [36], estrogens [37,38]) and ximelagatran [39]. Against this background, the mission of the EVITA evaluation tool is to focus strictly on the therapeutic benefit of the investigational drug. More importantly, EVITA compares the drug with other therapeutic approaches which are accepted as established therapy. Thus, EVITA does not evaluate the efficacy or efficiency of a new drug *per se*, but rather the potential superiority over a given established therapy. It should be noted that innovative value is also attributed by EVITA to a new pharmaceutical product, if it results - with similar efficiency - from a more favorable profile of adverse effects. EVITA comparisons of therapeutic principles are valid only for one and the same specific indication.

In the interests of a well-balanced comparison, adverse effect data is taken mainly from the same clinical studies as the data for the comparison of the investigational drug with the established therapy. Only if no satisfactory data is available, or if the compound has been on the market for a long time, is the adverse effect information for the investigational drug or the comparator extracted from the SPC.

An EVITA assessment, including all the elements from "therapeutic aim" to "EVITA score", is the evaluation of the additional therapeutic value of a new drug in a given indication. Attribution of the "N/A" assessment means that an EVITA calculation was not available due to insufficient data. This is, without any doubt, an assessment result. By gaining an N/A assessment, even more serious concerns are raised against a new drug regarding its potential contribution to the therapeutic choices in this indication. Moreover, the N/A result can be considered as a criticism passed on the methods to develop drugs and push them into the markets.

As is true for any health technology evaluation procedure, EVITA is characterized by obvious strengths but also weaknesses. A weakness might be that the NNTs for a given therapeutic approach are not always readily available from all the clinical trials, which is particularly true of studies using surrogate scale-scores as outcomes and of older compounds. Since EVITA will be further developed and validated for drugs which are immediately pre- or post-approval, the lack of NNT calculation will, however, be a rare rather than a frequent problem. In addition, EVITA, in the present version, does not allow weighting studies for methodological quality. This will hamper its applicability when numerous and methodologically different studies have already been performed. However, EVITA was developed for the assessment of new, recently approved compounds, i.e. when data from only a few clinical trials are available. One of the strengths of EVITA, on the other hand, is its transparency. Once established, EVITA evaluations will be published on the internet http://www.evita-report.de together with all clinical data upon which they are based. In addition, the evaluation *per se *is transparent, since all details such as positive and negative point scores and correction factors are clearly stated. The most important strength of EVITA is probably the fact that it can be easily and quickly adapted to the results of new clinical trials or even to a new interpretation of such data. This is especially important, since only a few clinical trials are available at the time of a drug's market entrance and its clinical relevance can often only be confirmed or denied by integrating new evidence into the evaluation process.

## Conclusions

EVITA has not been developed to replace established tools of comprehensive health technology assessment, since important methodological characteristics such as meta-analytical calculations are not included. It is rather intended to answer, in the sense of horizon scanning, the simple question of what a new drug will add to the therapeutic strategies already available for a given disease. The strict evaluation algorithm should stimulate, in addition, the discussion on the innovative value of new pharmacological compounds. In the next step of the project, the point scoring of both benefit and risk will be tested on larger numbers of newer and older pharmaceuticals both immediately and years after their market authorization to test the "forecast" value of EVITA. EVITA results will be presented on the internet to enhance professional debate about medical progress in pharmacotherapy.

## Competing interests

The authors declare that they have no competing interests.

## Authors' contributions

IP made substantial contributions to conception and design of the EVITA tool, to research and critical appraisal of trials, and to interpretation and assignment of data. She has been involved in drafting the manuscript and revising it critically for important intellectual content. NS made substantial contributions to conception and design of the EVITA tool, to critical appraisal of trials, and to interpretation of data. He has been involved in drafting and revising the manuscript and given approval of the version to be published. AM made substantial contributions to conception and design of the EVITA tool, to critical appraisal of trials, and to interpretation of data. He has been involved in drafting the manuscript. GL made substantial contributions to conception and design of the EVITA, with an especial focus on epidemiological issues. He has been involved in drafting the manuscript. BM made substantial contributions to conception and design of the EVITA tool, to critical appraisal of trials, and to interpretation and assignment of data to the tool. He has been involved in drafting the manuscript and revising it critically for important intellectual content and has given final approval of the version to be published. All authors read and approved the final manuscript.

## Pre-publication history

The pre-publication history for this paper can be accessed here:

http://www.biomedcentral.com/1472-6904/10/5/prepub
